# Reflections on Corporate Political Activity Taxonomies in the Context of Non-communicable Diseases and Mental Health: Comment on "Corporate Political Activity: Taxonomies and Model of Corporate Influence on Public Policy"

**DOI:** 10.34172/ijhpm.9124

**Published:** 2025-11-26

**Authors:** Téa Collins, Amanda Karapici

**Affiliations:** Department of Noncommunicable Diseases and Mental Health, World Health Organization, Geneva, Switzerland.

**Keywords:** NCDs, Unhealthy Commodity Industries, Multisectoral and Multistakeholder Action

## Abstract

This commentary examines Ulucanlar et al^1^ taxonomies on corporate political activity (CPA) through the lens of non-communicable diseases (NCDs) and mental health. While the study provided a useful framework for understanding harmful corporate strategies impacting public health, this commentary offers further insights into the applicability of these taxonomies to the NCD and mental health agendas. In addition, it proposes priorities for future research to explore how political ideologies and economic structures shape unhealthy commodity industry’s (UCI’s) influence on NCD policies, investigate industry funding of science and professional training, overcome the barriers to implementing the World Health Organization (WHO) "Best Buy" interventions,^2^ and improve governance mechanisms for multisectoral and multistakeholder coordination. This commentary underscores the importance of tailoring NCD policy responses to the unique challenges posed by UCIs while fostering accountable engagement with the broader private sector.

## Introduction

 Every two seconds, an individual under 70 dies from a non-communicable disease (NCD) at an age when they can be most productive.^3^ NCDs, such as cardiovascular diseases, cancers, chronic respiratory diseases and diabetes, accounted for at least 43 million deaths, representing 75% of non-pandemic-related global mortality.^3^ A disproportionate 73% of these NCD-related deaths occurred in low- and middle-income countries.^3^ Beyond mortality, NCDs and mental health conditions contributed to four out of every five years lived with a disability, with mental health disorders affecting close to 1 billion people worldwide and frequently remaining untreated.^3,4^ Key modifiable risk factors for NCDs include tobacco use, harmful alcohol consumption, unhealthy diets, and physical inactivity.

 The role of unhealthy commodity industries (UCIs), including those involved in tobacco, alcohol, ultra-processed foods, and gambling, has been pivotal in driving the burden of NCDs. Ulucanlar et al^1^ argue that UCIs, such as tobacco, alcohol, ultra-processed foods and beverages, and gambling are responsible for much of the disease burden attributable to NCDs. The authors propose a new model of cross-industry taxonomies to assist policy-makers and the broader public health community in critically evaluating corporate political activities (CPAs) of UCIs, which often prioritize business interests over public health. They conclude that strategies to counter CPA and protect public health policy are urgently needed.

 While the study provided a valuable framework for understanding harmful corporate strategies impacting public health, this commentary expands on the applicability of these taxonomies to NCD and mental health agendas. Additionally, it proposes future research priorities to explore how political ideologies and economic structures shape UCIs’ influence on NCD policies, investigating industry funding of science and professional training, overcoming barriers to implementing the World Health Organization (WHO) “Best Buy” interventions,^2^ and improving multistakeholder coordination.

## UCI Corporate Political Activity and Influence on NCD and Mental Health Agendas

 The influence of corporate interests on health, especially concerning NCDs and mental health conditions, has garnered significant attention since the United Nations General Assembly High-level Meeting on NCD Prevention and Control in 2011. UCIs, such as tobacco, alcohol, ultra-processed food, and fossil fuels, have been acknowledged as a driving force of NCDs and their shared risk factors.

 Ulucanlar et al^1^ focus on specific industries such as tobacco, alcohol, ultra-processed food, and gambling. However, the broader scope of how these industries exacerbate NCD risk factors through aggressive marketing and resistance to evidence-based policies, such as taxation and food labeling, remains underexplored.^5^ By articulating these connections more clearly, the authors could have presented a more comprehensive picture of how UCIs hinder policy measures aimed at addressing these risk factors. When combined with the WHO manual on fiscal policies,^6^ this would have reinforced the need to counter UCI influence more effectively.

 One notable gap in Ulucanlar et al discussion is the lack of focus on mental health. While gambling is acknowledged as a harmful industry, its effects are primarily felt through mental health pathways (ie, distress, financial hardship).^7^ Although the authors briefly note that “*many NCDs including mental health problems are caused by tobacco, ultra-processed foods, alcohol, and gambling,*” mental health is not adequately discussed throughout the paper. The inclusion of gambling without a broader consideration of mental health represents a missed opportunity to elaborate on how harms arising from commercial forces can affect nations’ mental health and well-being.

 Mental health conditions are increasingly recognized alongside NCDs in global health agendas due to multimorbidity and the chronic nature of these conditions. The 2030 Agenda for Sustainable Development Goals target 3.4 calls for a one-third reduction in premature mortality from NCDs through prevention and treatment and promoting mental health and well-being.^8^ The gambling industry is a clear example of commercial actors affecting mental health, deploying many of the same UCI strategies (sponsoring research, marketing tactics) to resist regulation of betting environments and advertising. Moreover, commercial practices of other industries, such as motor vehicles, mining, and fossil fuels, also negatively impact NCDs and health equity, but they were not included in the scope of the paper. In addition, social media and gaming have been linked with mental health conditions in young people.^4^

 Ulucanlar et al treat UCIs collectively due to the inherent harm their products cause. However, a more explicit differentiation between UCIs and other industries that influence NCDs would add clarity. For example, tobacco is singled out due to its conflict with public health objectives under the WHO Framework Convention on Tobacco Control, which mandates governments to protect public policy from tobacco industry interference.^9^ Collaboration with the alcohol industry, similarly, offers no clear public health benefits. By distinguishing between UCIs and other sectors, policy-makers and health agencies would be better equipped to engage with industries in ways that prioritize public health over business interests.

 Not all commercial actors oppose public health. Industries such as sports, information technology, and pharmaceuticals may play supportive roles in the prevention of NCDs and mental health conditions. These industries may require distinct approaches from governments to incentivize alternative sustainable business models conducive to making positive health, social and environmental impacts.

 Clear guidelines are needed to determine which industries should be excluded from health policy-making due to conflicts of interest and which ones should be included in the search for NCD solutions. In this regard, WHO has a clear framework in place for engaging with the private sector, such as the “*Framework of engagement with non-State actors.*”^10^ Additionally, WHO has developed a practical tool for informed decision-making to assist Member States in engaging with private sector entities for the prevention and control of NCDs while managing potential conflicts of interest.^11^ These tools promote a principle-based vetting of private entities to ensure credibility and protect policies from vested interests and ensure compatibility with health and equity objectives. By explicitly acknowledging different industries, future CPA frameworks can better guide policy-makers in managing engagements with relevant industries by anticipating and countering undue industry interference in public policy.

## Research Agenda for Engaging With the Private Sector for NCD Prevention and Control and Mental Health

 Ulucanlar et al call for identifying and evaluating the best approaches and interventions to address CPA and reverse the growing burden of NCDs. We propose several priority research areas for potential solutions:

1. *Countries’ political economy and prevailing ideologies influencing the political activities of UCIs*. Political economy analysis can explore the role of the industry in low- and middle-income countries versus high-income countries, to understand the context-specific political, economic, social, cultural, and scientific systems, as Ulucanlar et al note. Additionally, some authors argue that the dominance of neoliberal ideologies, which emphasize deregulation, free markets, and limited government intervention, can create an enabling environment for UCIs to thrive, becoming structural barriers to NCD prevention policies.^12^ Thus, research could investigate how different political systems and ideologies are associated with the extent of industry interference and how it affects the adoption of NCD policies.
2. *Financing mechanisms employed by UCIs to exert their influence.* Another critical area for inquiry is understanding the financing mechanisms used by UCIs. Ulucanlar et al taxonomy touches on how corporations “shape evidence to manufacture doubt,” including providing funding to bias the research evidence, sponsoring events to create the illusion of corporate social responsibility or advertising widely unhealthy foods and beverages, including to school children.^13^ Some research priorities may include the need to understand UCIs’ strategies for funding research institutions, continued education, and professional associations; industry efforts to influence medical and public health professionals to minimize the importance of prevention policies; and industry resource contributions to professional organizations that tamper with their advocacy efforts to scale up action for NCD prevention and control. Addressing these issues would help identify potential conflicts of interest undermining health systems’ efforts to address NCDs, their shared risk factors and mental health conditions. Conflict of interest disclosures and developing codes of conduct for health organizations may help to address these challenges.^11^ More empirical evidence on industry financing patterns could inform stricter guidelines leading to greater transparency and perhaps new governance mechanisms that might counteract the industry’s financial influence on policy development, knowledge generation and training.
3. *Industry interference in the implementation of the “Best Buy” interventions to address NCDs and mental health conditions.* The WHO has promoted a set of “Best Buys” cost-effective interventions to address NCDs and their risk factors.^2^ Despite broad consensus on these interventions, many countries struggle to adopt and implement them fully due to political and commercial factors,^14^ rather than technical feasibility. The work of Ulucanlar et al is directly relevant here, as it documents the strategies industries use to deter or weaken exactly these kinds of policies. Research is needed to delve into country-level experiences with implementing “Best Buys,” identifying how industry opposition has manifested itself and how some governments have overcome it. Cross-country comparative studies, as well as an analysis of multilevel governance systems that promote public interests over business profits, alternative sustainable business models creating social values, and exploration of how an investigation of strong regulatory frameworks might protect health policies, can shed light on these questions. Implementation science approaches that incorporate political will and industry interference as key variables will be especially useful. Documenting successes and failures can guide countries and create a playbook for navigating the interference that Ulucanlar et al taxonomy so clearly articulates.
4. *Policy coherence and multisectoral and multistakeholder approaches.* Effective NCD prevention and control and addressing mental health challenges requires a “whole-of-society” and “whole-of-government” response and multisectoral policies that will counter commercial practices. All government sectors, civil society, academia, professional organizations, the private sector, and people living with or at risk of these conditions, all have a role to play in bringing about policy change, pooling resources and generating innovative solutions.^15^ A research path will explore models of multistakeholder engagement that have succeeded or failed in addressing NCDs and endeavor to understand how conflicts of interest were navigated in these scenarios for achieving public health objectives.

## Conclusion

 Ulucanlar et al^1^ provide an appealing taxonomy for understanding how UCI seeks to influence and undermine public health policy. This commentary considers CPA taxonomies in the context of NCD prevention, control, and mental health, identifying critical gaps and proposing research directions to enhance both practice and research. Future studies should sharpen the focus on specific NCDs and mental health risk factors while ensuring a clear distinction between harmful industry entities and those that can contribute positively. Additionally, developing strategies to navigate the political economy that enables corporate interference is crucial and sheds light on financing mechanisms. Lastly, it also calls for innovative governance approaches to implement the WHO Best Buys and requires a “whole-of-society” and “whole-of-government” response under principles that prioritize public health over commercial profit.

## Disclosure of artificial intelligence (AI) use

 Not applicable.

## Ethical issues

 Not applicable.

## Conflicts of interest

 Authors declare that they have no conflicts of interest.

## Disclaimer

 The authors are staff members of the WHO. The authors alone are responsible for the views expressed in this publication, and they do not necessarily represent the views, decisions, or policies of the WHO.

## References

[1] Ulucanlar S, Lauber K, Fabbri A (2023). Corporate political activity: taxonomies and model of corporate influence on public policy. Int J Health Policy Manag.

[2] World Health Organization (WHO). Tackling NCDs: Best Buys and Other Recommended Interventions for the Prevention and Control of Noncommunicable Diseases. 2nd ed. WHO; 2024.

[3] United Nations. Progress on the Prevention and Control of Non-Communicable Diseases and the Promotion of Mental Health and Well-Being: Note by the Secretary-General (A/79/732). United Nations; 2025. https://docs.un.org/en/A/79/762.

[4] World Health Organization (WHO). World Mental Health Report: Transforming Mental Health for All. WHO; 2022. https://iris.who.int/handle/10665/356119.

[5] Tangcharoensathien V, Chandrasiri O, Kunpeuk W, Markchang K, Pangkariya N (2019). Addressing NCDs: challenges from industry market promotion and interferences. Int J Health Policy Manag.

[6] World Health Organization (WHO). Fiscal Policies to Promote Healthy Diets: WHO Guideline. WHO; 2024. https://iris.who.int/handle/10665/376763. 38976625

[7] Badji S, Black N, Johnston DW (2023). Economic, health and behavioural consequences of greater gambling availability. Econ Model.

[8] United Nations. Transforming Our World: The 2030 Agenda for Sustainable Development. United Nations; 2015.

[9] World Health Organization (WHO). WHO Framework Convention on Tobacco Control. WHO; 2003. https://iris.who.int/handle/10665/42811.

[10] World Health Organization (WHO). Framework of Engagement with Non-State Actors. WHO; 2016.

[11] World Health Organization (WHO). Supporting Member States in Reaching Informed Decision-Making on Engaging with Private Sector Entities for the Prevention and Control of Noncommunicable Diseases: A Practical Tool. WHO; 2024. https://iris.who.int/handle/10665/378209.

[12] Lencucha R, Thow AM (2019). How neoliberalism is shaping the supply of unhealthy commodities and what this means for NCD prevention. Int J Health Policy Manag.

[13] Collins TE, Akselrod S, Mahy L (2023). Engaging with the private sector for noncommunicable disease prevention and control: is it possible to create “shared value?”. Ann Glob Health.

[14] Loffreda G, Arakelyan S, Bou-Orm I (2024). Barriers and opportunities for WHO “Best Buys” non-communicable disease policy adoption and implementation from a political economy perspective: a complexity systematic review. Int J Health Policy Manag.

[15] Collins T, Mikkelsen B, Axelrod S (2019). Interact, engage or partner? Working with the private sector for the prevention and control of noncommunicable diseases. Cardiovasc Diagn Ther.

